# The Risk of QTc-Interval Prolongation in Breast Cancer Patients Treated with Tamoxifen in Combination with Serotonin Reuptake Inhibitors

**DOI:** 10.1007/s11095-019-2746-9

**Published:** 2019-12-16

**Authors:** Koen G. A. M. Hussaarts, Florine A. Berger, Lisette Binkhorst, Esther Oomen - de Hoop, Roelof W. F. van Leeuwen, Robbert J. van Alphen, Daniëlle Mathijssen - van Stein, Natasja M. S. de Groot, Ron H. J. Mathijssen, Teun van Gelder

**Affiliations:** 1 Department of Medical Oncology, Erasmus MC Cancer Institute, Erasmus University Medical Center, Doctor Molewaterplein 40, PO Box 2040, 3015 GD Rotterdam, The Netherlands; 2 Department of Hospital Pharmacy, Erasmus MC, Erasmus University Medical Center, Rotterdam, the Netherlands; 3 Department of Hospital Pharmacy, HAGA Hospital, Den Haag, the Netherlands; 4 Department of Internal Medicine, Elisabeth-Tweesteden hospital, Tilburg, the Netherlands; 5 Department of Internal Medicine, Franciscus Vlietland & Gasthuis, Schiedam, the Netherlands; 6 Department of Cardiology, Erasmus MC, Erasmus University Medical Center, Rotterdam, the Netherlands

**Keywords:** Tamoxifen, serotonin reuptake inhibitors, drug-drug interaction, electrocardiogram, QTc-interval

## Abstract

**Purpose:**

Antidepressants like the serotonin reuptake inhibitors (SRIs) are often used concomitantly with tamoxifen (e.g. for treatment of depression). This may lead to an additional prolongation of the QTc-interval, with an increased risk of cardiac side effects. Therefore we investigated whether there is a drug-drug interaction between tamoxifen and SRIs resulting in a prolonged QTc-interval.

**Methods:**

Electrocardiograms (ECGs) of 100 patients were collected at steady state tamoxifen treatment, with or without concomitant SRI co-medication. QTc-interval was manually measured and calculated using the Fridericia formula. Primary outcome was difference in QTc-interval between tamoxifen monotherapy and tamoxifen concomitantly with an SRI.

**Results:**

The mean QTc-interval was 12.4 ms longer when tamoxifen was given concomitantly with an SRI (95% CI:1.8–23.1 ms; *P* = 0.023). Prolongation of the QTc-interval was particularly pronounced for paroxetine (17.2 ms; 95%CI:1.4–33.0 ms; *P* = 0.04), escitalopram (12.5 ms; 95%CI:4.4–20.6 ms; *P* < 0.01) and citalopram (20.7 ms; 95%CI:0.7–40.7 ms; *P* = 0.047), where other agents like venlafaxine did not seem to prolong the QTc-interval. None of the patients had a QTc-interval of >500 ms.

**Conclusions:**

Concomitant use of tamoxifen and SRIs resulted in a significantly higher mean QTc-interval, which was especially the case for paroxetine, escitalopram and citalopram. When concomitant administration with an SRI is warranted venlafaxine is preferred.

## Introduction

One of the most common causes of cessation of therapeutic use of drugs which have already been marketed is prolongation of the QT-interval, which is defined as a QT interval > 470 ms in females and > 450 ms in males according to European Society of Cardiology (ESC) guidelines (1,2). QT-interval or the heart-rate corrected QT (QTc) interval prolongation is associated with higher risk of polymorphic ventricular tachycardia or Torsade des Pointes (TdP), which may ultimately lead to sudden cardiac death (SCD) (1,3). The QTc-interval represents the duration between the onset of ventricular depolarization and the completion of repolarization of the myocardium. Several risk factors are associated with an increased risk for QTc-interval prolongation (e.g hypopotassemia, renal impairment, use of diuretics and other QTc-prolonging drugs and unmodifiable risk factors such as age > 65 years and female gender) (1,4–6). Furthermore, it has become evident that several classes of anti-cancer drugs are associated with QT prolongation and, therefore, this offers a great challenge in the treatment of cancer patients (7–9).

**Fig. 1:**
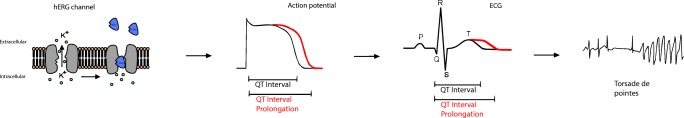
Mechanism of QTc-interval prolongation. Serotonin reuptake inhibitors (SRIs) inhibit the hERG channel and therefore the I_kr_ (repolarizing potassium (K^+^) current) in the cardiomyocyte. This results in a delay of the ventricular repolarization time and therefore in a prolongation of the QTc-interval. Prolongation of the QTc-interval may result in cardiac arrhythmias such as TdP.

The suggested mechanism of drug-induced QTc-interval prolongation is inhibition or reduced expression of the Human ether-a-go-go related (hERG) gene that encodes a potassium channel that regulates repolarizing currents (I_kr_) in the cardiomyocytes or inhibition of late sodium currents (1,10). Inhibition of these I_kr_ results in a delay in the ventricular repolarization causing prolongation of the QT-interval (Fig. 1). Some drugs are known I_kr_ inhibitors, but failed to demonstrate a clinical significant QTc-interval prolongation at dosages used in routine clinical practice (e.g. fexofenadine), although some of these drugs still give an increased risk of experiencing TdP (1). Therefore, the risk of experiencing TdP is not fully linear with the extent of QTc-interval prolongation. Combining QTc-prolonging drugs (drug-drug interaction) may also increase the risk of SCD (1,8). The combination of two known QTc-prolonging drugs may result in a cumulative or synergistic prolongation of the QTc-interval and thus increased risk for TdP (11,12).

The risk of drug-induced QTc-interval prolongation is determined according to Adverse Drug Event Causality Analysis into QTc-prolonging drugs with a ‘known’, ‘possible’ or ‘conditional’ risk for TdP (13,14). A drug is categorized as a drug ‘with a known risk of TdP’ if there is substantial positive evidence of prolongation of the QTc-interval and an association with TdP. The risk is scored as ‘possible’ if there is substantial evidence which supports the conclusion that drugs can prolong the QTc interval, but there is insufficient evidence that these drugs are associated with TdP. Finally, the risk is scored as ‘conditional’ if there is substantial evidence of QT-interval prolongation with an association with TdP development but only under certain conditions (e.g. overdosing) or because the drug has shown ability to create one or more conditions that facilitate induction of TdP (e.g. by inhibiting metabolism of QTc-proloning drugs). Drugs with a ‘known’ risk for QTc-interval prolongation are escitalopram and citalopram. Venlafaxine, imipramine, nortriptyline and tamoxifen are classified as ‘possible’ and paroxetine, amitriptyline, sertraline, fluoxetine and fluvoxamine are classified as conditional according to Crediblemeds®.

One of the anti-cancer drugs, which is a known I_kr_ inhibitor, is the selective ER modulator (SERM) tamoxifen (8,15,16). Since decades, tamoxifen is used in the treatment of breast cancer, where it provides suppression of ER-dependent proliferation of breast cancer cells and therefore reduces the risk of disease recurrence and mortality. However, tamoxifen may also lead to I_kr_ inhibition in cardiac tissue and ultimately to prolongation of the QTc-interval (15). After absorption tamoxifen is converted into several pharmacologically active metabolites of which endoxifen is the most potent. The cytochrome P450 enzymes CYP2D6 and CYP3A4 play a dominant role in the biotransformation of tamoxifen (17). It has been shown that the use of CYP2D6 or CYP3A4 inhibitors or inducers may lead to a significant alteration in tamoxifen and endoxifen exposure (18–20). One of the classes of drugs that is known for its ability to inhibit CYP2D6 are the serotonin reuptake inhibitors (SRIs) like the selective serotonin reuptake inhibitors (SSRI) and the serotonin norepinephrine reuptake inhibitor (SNRI) venlafaxine (21). These drugs are frequently used (by breast cancer patients) for the treatment of depression, anxiety disorders or (tamoxifen-related) hot flashes (22). The most potent CYP2D6-inhibiting SRIs are paroxetine and fluoxetine (21). When coadministration of an SRI is necessary with tamoxifen therapy, patients are often treated with weak CYP2D6-inhibiting SRIs like citalopram or escitalopram to minimize the risk of changes in endoxifen plasma concentrations (19,20). However, SRIs such as citalopram and escitalopram are also known to cause prolongation of the QTc-interval (23).

Since both tamoxifen and SRIs may prolong the QTc-interval, the combined use of these drugs may result in an enhanced risk of prolongation of the QTc-interval and therefore ventricular arrhythmias, especially in breast cancer patients since they have often more additional risk factors (e.g. female gender, often older age). At present it is unknown if the effect of combined treatment on the QTc-interval is additive or synergistic. Hence, the objective of this study was to determine whether there is a clinically relevant drug-drug interaction between tamoxifen and SRIs resulting in a prolonged QTc-interval.

## Materials and Methods

### Study Design

This observational study was performed between February 2012 and October 2018. Electrocardiogams (ECGs) were collected in the Erasmus University Medical Center in Rotterdam, the Franciscus Vlietland & Gasthuis in Schiedam and the Elisabeth-Tweesteden hospital in Tilburg, the Netherlands. This study has focused on the QTc-interval during treatment with tamoxifen monotherapy compared to treatment with tamoxifen and SRIs (i.e. SSRIs, SNRIs and tricyclic antidepressants). The study was approved by the local ethics committee of the Erasmus Medical Center in Rotterdam (MEC12–109).

### Study Population

We included a total of 100 adult patients with breast cancer for whom treatment with tamoxifen was indicated. Fifty patients also used an SSRI, venlafaxine or a tricyclic antidepressant, which also inhibits serotonin reuptake and may increase the risk for QTc-interval prolongation (e.g. amitriptyline). ECGs were taken at any time interval following drug intake. Patients were on tamoxifen treatment for at least 4 weeks. Patients were included either retrospectively or prospectively. Patients should not have received chemotherapy or radiotherapy within 4 weeks prior to the ECG-recording. If patients were included prospectively, written informed consent was obtained. If the ECG of patients showed a left or right bundle branch block (LBBB/RBBB), atrial fibrillation or other ECG abnormalities due to cardiac pathology, ischaemia or bigeminy, they were excluded from further analysis owing to interference of these factors with the QTc-interval. ECGs showing a QRS complex of >120 ms, RR intervals >1800 ms (defined as the time between two consecutive R waves) or < 500 ms or ECGs with a QTc interval > 700 ms or < 300 ms were also excluded, since the QTc-interval could not be reliably measured. In addition, patients who used other strong inhibitors/inducers of CYP2D6 and/or CYP3A4 (according to the Flockhart table) were excluded from the analysis (21). Medication with a ‘known’ risk of TdP according to the *CredibleMeds list* of QTc-prolonging drugs, except for tamoxifen and SRIs, was prohibited and considered as exclusion criterion (13).

### Outcome Measures and Data Collection

The primary outcome measure of this study was the difference in QTc-interval duration between tamoxifen monotherapy and tamoxifen therapy with concomitant use of SRIs. Secondary outcomes were the difference in the prevalence of QTc-interval prolongation between the two groups and the identification of risk factors for QTc-interval prolongation. QTc-interval prolongation was defined as a QTc-time of >470 ms in females and > 450 ms in males, based on the ESC guidelines (2). Twelve-lead ECGs were recorded and QT-intervals were measured manually by the same researcher for all patients, preferably from lead II, from the onset of the QRS complex to the end of the T-wave, according to the tangent method, and were corrected for heart rate using the Fridericia formula (QTcF) (24). The Fridericia formula is formulated as the QT-interval divided by the RR-interval to the power 0.33 (QTcB = QT/(RR^0.33^)) (25). For each patient data on characteristics such as age, sex, medical history, tumor localization, previous anti-cancer treatment, laboratory analysis (i.e. liver function [AST, ALT, bilirubin], renal function [creatinin, glomerular filtration rate(eGFR)], electrolytes [sodium, potassium, calcium, magnesium]) and medication was obtained from electronic patient records (HIX, Chipsoft b.v., Amsterdam, the Netherlands). ECGs were obtained during tamoxifen or tamoxifen concomitant with an SRI therapy, when steady state therapy for both therapies was reached (determined as at least four weeks of use for tamoxifen and one week for SRIs). A baseline ECG was determined as an ECG before start of tamoxifen or SRI therapy.

### Statistical Analysis

QTc-intervals were compared between patients receiving tamoxifen monotherapy and patients receiving tamoxifen with concomitant SRI therapy. To detect a difference of 15 ms, assuming a standard deviation for QTc-interval time of 26 ms, in mean QTc-interval between both groups with 80% power, a total of one hundred patients was required. Therefore, a total of fifty evaluable patients using tamoxifen monotherapy and fifty evaluable patients using tamoxifen concomitant with an SRI were included in the study. A *p* value ≤0.05 was considered statistically significant. Data was analyzed using IBM SPSS Statistics version 24 (IBM Corporation, Armonk, NY). A *t*-test for independent samples was used to compare the mean QTc-interval between the treatment groups. Furthermore, difference between treatment groups in mean age was also determined using a *t-*test. For the other patient characteristics the chi-square test was used. Moreover for age, renal function, sodium, potassium, calcium and magnesium a Pearson correlation coefficient was estimated to determine the correlation with the QTc-interval. Correlation for other parameters as tumor localization and previous therapy (e.g. anthracyclines, trastuzumab and radiotherapy) was estimated using a Spearman correlation coefficient. For the secondary outcome the QTc-interval was dichotomized as either prolonged if >470 ms for females or not prolonged if otherwise, according to the ESC guidelines (2). Difference in proportion of QT-interval prolongation between groups was determined using the Fisher’s exact test. Univariate logistic regression analysis was performed to determine associated risk factors. If there were any significant risk factors they were put into a multivariate analysis.

## Results

### Participants

A total of 111 breast cancer patients were initially included in this study. Eleven patients were excluded due to a variety of ECG abnormalities at baseline resulting in a total of 100 evaluable patients. Fifty patients were treated with tamoxifen in combination with an SRI (further referred to as index group) and 50 patients were treated with tamoxifen without an SRI (further referred to as control group). All patients were female. The median age of patients in the control group (60; interquartile range (IQR) = 50–66 years) was significantly higher than the median age of patients in the index group (50; IQR = 45–59 years; *P* = 0.01). There were no other statistically significant differences between the two groups and none of the patients experienced cardiac arrhythmias. The most frequently used SRIs in the index group were venlafaxine (30%) and paroxetine (20%). A more detailed overview of the patient characteristics is presented in Table I.

**Table I:** Patient characteristics

Characteristic	Index group (*N* = 50) (%)	Control group (*N* = 50) (%)	*P* value of the difference
Age (Median, IQR)*	50 (45–59)	60 (50–66)	0.01*
- < 65 years	41 (82)	37 (74)	
- ≥ 65 years	9 (18)	13 (26)	
Sex			NA
- Female	50	50	
Race			0.28
- Caucasian	45 (90)	45 (90)	
- Arabic	4 (8)	1 (2)	
- African	1 (2)	1 (2)	
- Latino	–	3 (6)	
Breast cancer localization^#^			0.54
- Left	25 (50)	22 (44)	
- Right	23 (46)	28 (56)	
Trastuzumab pretreatment			0.20
- Yes	8 (16)	3 (6)	
- No	42 (84)	47 (94)	
Anthracycline pretreatment			1.0
- Doxorubicin	21 (42)	17 (34)	
- Epirubicin	19 (38)	22 (44)	
- No	10 (20)	11 (22)	
Radiotherapy			0.69
- Yes	26 (52)	29 (58)	
- no	24 (48)	21 (42)	
Number of drugs	2 (0–6)	2 (0–4)	0.93
Tamoxifen dose			0.20
20 mg	45 (90)	49 (98)	
40 mg	5 (10)	1 (2)	
Type of antidepressant			NA
- Venlafaxine	15 (30)		
- Paroxetine	10 (20)		
- Escitalopram	5 (10)		
- Citalopram	5 (10)		
- Amitriptyline	5 (10)		
- Sertraline	4 (8)		
- Fluoxetine	3 (6)		
Other	3 (6)		
Renal dysfunction	1	3	0.30
Electrolyte disturbances			
- Hyponatremia	2	0	0.50
- Hypopotassemia	0	0	–
- Hypocalcemia	3	2	1.0
- Hypomagnesemia	1	2	1.0
Hepatic dysfunction	1	1	1.0
Antidiabetic use	4 (8)	3 (6)	1.0
Loopdiuretic use	0 (0)	1 (2)	1.0

*Abbrevations*: *IQR* interquartile range, *NA* not applicable, Other type of antidepressant were fluvoxamine (*n* = 1), imipramine (*n* = 1) and nortriptyline (*n* = 1). Renal dysfunction was defined as estimated Glomerular Filtration Rate (eGFR) < 60 m/min/1,73m^2^, Hyponatremia was defined as a sodium value < 136 mmol/l, Hypopotassemia was defined as a potassium value < 3.5 mmol/l, hypocalcemia was defined as a calcium value < 2.2 mmol/l, hypomagnesemia was defined as a magnesium value < 0.7 mmol/l and hepatic dysfunction was defined as increased bilirubin (>16umol/l), increased alanine aminotransferase (ALAT) (>40 U/l) or increased aspartate transaminase (ASAT) (>35 U/l). Missing values: Hepatic function (*N* = 33), Sodium (*N* = 31), potassium (*N* = 27), calcium (*N* = 60), magnesium (*N* = 66) and renal function (*N* = 28); * = P value < 0.05. ^#^ For breast cancer localization the equation was made for left or right. There was 1 patient with breast cancer on both sides at primary diagnosis and 1 patient for which data regarding tumor localization was unknown due to lack of information from the referring center. These patients were both excluded from this analysis

### Primary Outcome Measures

Mean QTc interval was 407.5 ± 22.1 ms in the control group and 419.9 ± 24.1 ms in the index group. This resulted in a significant difference in mean QTc interval of 12.4 ms (95%CI 1.8–23.1 ms; *P* = 0.023) (Table II). Heart rate was not significantly different between the control group and the index group.

**Table II:** QTc interval

	Mean QTc (Fridericia) time (ms) ± SD	Patients with QTc prolongation	Mean Difference (ms) (95%CI)	P value	Mean Heart rate (beats/min) ± SD
Tamoxifen monotherapy	407.5 ± 22.1	1 (2%)			70 ± 13.6
Tamoxifen with SSRI	419.9 ± 24.1	0 (0%)^#^	+12.4 (1.8 to 23.1)	P = 0.023*	69 ± 10.9
- Venlafaxine	408.8 ± 21.5		+1.3 (−11.4 to 14.0)	0.84	
- Paroxetine	424.7 ± 29.2		+17.2 (1.4 to 33.0)	0.04*	
- Escitalopram	420.0 ± 6.0		+12.5 (4.4 to 20.6)	< 0.01	
- Citalopram	428.2 ± 16.6		+20.7 (0.7 to 40.7)	0.047*	
- Amitriptyline	428.8 ± 32.5		+21.3 (−0.1 to 42.5)	0.05	
- Sertraline	424.3 ± 24.1		+17.0 (−5.6 to 39.6)	0.15	
- Fluoxetine	414.7 ± 25.6		+7.2 (−18.7 to 33.1)	0.59	

*Legend*: This table shows the QTc times and difference in QTc-interval between treatment groups. * *P* value <0.05. For the analysis of the differences an independent samples t-test was used. ^#^Difference in number of patients with QTc prolongation was not significant (*P* = 1.0)

### Secondary Outcome Measures

Analysis with the Fridericia formula resulted in 1 patient with a prolonged QTc-interval, which was in the control group. This resulted in a prevalence of 2% in the control group and a prevalence of 0% in the index group, which was a non-significant difference (*P* = 1.0). None of the patients had a QTc-interval of >500 ms. SRI subgroup analysis showed a significant difference in mean QTc-interval time for paroxetine (17.2 ms; 95%CI 1.4–33.0 ms; *P* = 0.04), escitalopram (12.5 ms; 95%CI 4.4–20.6 ms; P = <0.01) and citalopram (20.7 ms; 95%CI 0.7–40.7 ms; *P* = 0.047) compared to the control group in contrast to the other SRIs, which did not show a significant difference in QTc-interval (Table II).

For the known risk factors for QTc-interval prolongation, only SSRI use (Spearman r = 0.25; *P* = 0.01), age (Pearson r = 0.24; *P* = 0.02), plasma potassium levels (Pearson r = −0.28, P = 0.02), renal dysfunction (Pearson r = −0.24; *P* = 0.04) and the use of >1 concomitant drugs used (Spearman r = 0.23, P = 0.02) showed significant correlation with QTc-interval duration. There were no other factors which showed a significant correlation with QTc-interval in general (Table III). Furthermore possible risk factors as tumor localization (left vs. right) and (pre)treatment with anthracyclines, radiotherapy or trastuzumab did not show statistically significant correlation with QTc-interval in general. Univariate analysis did not reveal any significant risk factors and therefore a multivariate analysis was not performed. The odds ratios for the individual risk factors could not be measured reliably, since the prevalence of QTc prolongation was low.

**Table III:** Risk factors for QTc-interval prolongation

Patients	QTc-interval prolongation (N = 1)	Correlation coefficient (*P* value)
Age		0.24 (0.02)*
Age > 65	1	0.18 (0.07)
Race		0.07 (0.47)
- Caucasian	0	
- Arab	0	
- African	0	
- Latino	1	
Breast cancer localization*		−0.16 (0.11)
- Left	1	
- right	0	
Trastuzumab	0	−0.03 (0.81)
Anthracyclines		0.14 (0.15)
- Doxorubicin	0	
- epirubicin	0	
- No		
Radiotherapy	1	0.10 (0.34)
Use of > 1 concomitant drug	1	0.23 (0.02)*
SRI use	0	0.25 (0.01)*
Type of SRI		0.27 (0.06)
- Venlafaxine	0	
- Paroxetine	0	
- Escitalopram	0	
- Citalopram	0	
- Amitriptyline	0	
- sertraline	0	
- Fluoxetine	0	
- Other	1	
Renal dysfunction	1	−0.24 (0.04)*
Electrolyte disturbances		
- Hyponatremia	0	−0.19 (0.12)
- Hypopotassemia	0	−0.28 (0.02)*
- Hypocalcemia	0	−0.14 (0.39)
- Hypomagnesemia	0	0.29 (0.09)
Hepatic dysfunction	0	0.20 (0.10)
Antidiabetics	1	−0.12 (0.24)
Loop diuretics	0	−0.10 (0.32)

*Legend*: Number of patients which show QTc-interval prolongation (QTc > 470 ms), when using the Fridericia formula. Furthermore the correlation coefficient was calculated and displayed. For breast cancer localization the equation was made for left or right. There was 1 patient with breast cancer on both sides at primary diagnosis and 1 patient for which data regarding tumor localization was unknown due to lack of information from the referring center. These patients were both excluded from this analysis. * *P* value<0.05

## Discussion

To our knowledge, this is the first study that investigated the additional risk of developing QTc- prolongation in patients using tamoxifen in combination with an SRI. This study showed a significant difference in the mean QTc-interval between patients treated with tamoxifen monotherapy and patients treated with tamoxifen therapy concomitantly with an SRI, suggesting an additional QTc-prolonging effect if tamoxifen is combined with an SRI. Furthermore, in this study 1% of the patients had a prolonged QTc-interval (>470 ms). This prevalence is in line with other clinical findings and a recent investigation in cancer patients treated with conventional or targeted anti-cancer therapy (26,27).

In this study, ECGs were retrospectively or prospectively collected during tamoxifen steady-state monotherapy or tamoxifen therapy combined with an SRI. One of the main limitations of this study was the absence of a baseline measurement in most of the patients. Therefore, a ‘change from baseline’ analysis could not be performed. There was a significant difference in mean QTc-interval time between the tamoxifen monotherapy and tamoxifen with SRI treated patients, which is most likely related to the additive effect of the SRI. As mentioned earlier tamoxifen is an assumed QTc-interval prolonging agent, especially in higher doses (8,16). Furthermore there is substantial evidence regarding QTc-interval prolongation by SRIs, showing an average increase in QTc-interval of 10-20 ms. QTc-interval prolonging effects seem most prominent in nortriptyline and citalopram with increases of more than 30 ms (28,29). Therefore an additive effect of SRIs seems possible on top of the QTc-interval prolonging effects of tamoxifen. However, to determine whether the use of an SRI in combination with tamoxifen is a significant/clinically relevant factor influencing the QTc-interval, more research is needed in patients having both a baseline ECG during tamoxifen use and at least a second ECG where tamoxifen is used in combination with an SRI.

Interestingly, a subgroup analysis of the different SRIs showed a significant increase of the QTc-interval for citalopram, escitalopram and paroxetine, which is in line with the classification on the *CredibleMeds* list. In this list, citalopram and also escitalopram has been clearly associated with QTc-interval prolongation. On this list paroxetine is classified as a drug which gives a ‘conditional’ risk of TdP. Several additional factors like antidiabetic drug use, renal dysfunction and multiple drug use may have contributed to QTc-interval prolongation in some of these patients. Furthermore, patients in the control group were significantly older than in the index group. The QTc-interval increases with age, and therefore in elderly patients, the criteria for QTc-interval prolongation will be met more frequently in the index group. We do acknowledge that due to limited sample size in the subgroup our study was underpowered to make definitive conclusions regarding individual drugs.

Although QTc-interval prolongation is carefully investigated during early drug development, its actual influence on overall survival remains unclear. It is clear that QTc-interval prolongation can lead to ventricular tachyarrhythmias (e.g. TdP) and SCD (1,3). A recent systematic review from Arunachalam et al. showed that ventricular tachyarrhythmias were observed in 2.6% of patients using QTc-interval prolonging drugs, however TdP (0.33%) and SCD (0.03%) were relatively rare (27). Since the absolute risk of cardiac events is small, physicians always need to weigh the benefits of cessation of a QTc-interval prolonging drug to the disadvantage of discontinuation of a potentially useful drug. If a QTc-interval prolonging drug can be replaced by a non QTc-interval prolonging agent, this should always be considered.

The interaction investigated in this study may be explained at a pharmacodynamic or pharmacokinetic level. Both tamoxifen and SRIs inhibit the I_kr_ and therefore, both may prolong the QTc-interval. Inhibition of CYP2D6 by SRIs results in lower endoxifen plasma levels (especially for strong CYP2D6 inhibitors like paroxetine) and possible more I_kr_ inhibition, because of the higher tamoxifen plasma levels. However preclinical evidence suggests similar I_kr_-inhibition by both tamoxifen and its metabolites, making this a less likely explanation (20,30–32). SRIs like fluoxetine and paroxetine are well known strong CYP2D6 inhibitors, which could alter endoxifen concentrations and deprive patients from optimal oncologic therapy. Escitalopram, citalopram and venlafaxine are weak CYP2D6 inhibitors and therefore are considered safe when administered concomitantly with tamoxifen (20). However, since escitalopram and citalopram are also ‘known’ QTc-interval prolonging drugs, the combination with tamoxifen is not desirable and venlafaxine may be a better alternative since it seems to prolong the QTc-interval in only a minor extent, as was shown in this study (Table II). However more research is needed to verify this point.

In conclusion, this study is the first clinical study that investigated the additional risk of QTc-interval prolongation in patients using an SRI concomitantly with tamoxifen. There was a significantly longer mean QTc-interval in the patients who used an SRI, which tended to be most prominent in patients receiving citalopram, escitalopram or paroxetine. The other SRIs, like venlafaxine and fluvoxamine, were not clearly associated with QTc-interval prolonging effects. Based on our data we recommend avoiding citalopram, escitalopram and paroxetine in tamoxifen treated women, and use the others SRIs that do not have this QTc-prolonging effect (e.g. venlafaxine and fluvoxamine) to minimize the possible risk of TdP and cardiac arrhythmias. As the degree of QTc-interval prolongation was limited, and none of the patients in this study reached a QTc-interval of >500 ms, routinely checking ECGs in patients on combined tamoxifen+SRI treatment does not seem necessary. For patients who have multiple other risk factors for QTc-interval prolongation and are using paroxetine, escitalopram and citalopram checking the QTc-interval duration may increase patient safety.

## Abbreviations

ECG: Electrocardiogram
ER: Estrogen receptor
ESC: European Society of Cardiology
hERG: Human ether-a-go-go related
I_kr_: Repolarizing potassium currents
IQR: Interquartile range
LBBB: Left bundle branch block
QTc: Heart-rate corrected QT interval
RBBB: Right bundle branch block
SCD: Sudden cardiac death
SNRI: Serotonin norepinephrine reuptake inhibitor
SRI: Serotonin reuptake inhibitor
SSRI: Selective serotonin reuptake inhibitor
TdP: Torsade des pointes
